# Contemporary Clinical Management of Endometrial Cancer

**DOI:** 10.1155/2013/583891

**Published:** 2013-06-24

**Authors:** Helen E. Dinkelspiel, Jason D. Wright, Sharyn N. Lewin, Thomas J. Herzog

**Affiliations:** Division of Gynecologic Oncology, Department of Obstetrics and Gynecology, Columbia University College of Physicians and Surgeons, 161 Fort Washington Avenue, HIP 8-838, New York, NY 10032, USA

## Abstract

Although the contemporary management of endometrial cancer is straightforward in many ways, novel data has emerged over the past decade that has altered the clinical standards of care while generating new controversies that will require further investigation. Fortunately most cases are diagnosed at early stages, but high-risk histologies and poorly differentiated tumors have high metastatic potential with a significantly worse prognosis. Initial management typically requires surgery, but the role and extent of lymphadenectomy are debated especially with well-differentiated tumors. With the changes in surgical staging, prognosis correlates more closely with stage, and the importance of cytology has been questioned and is under evaluation. The roles of radiation in intermediate-risk patients and chemotherapy in high-risk patients are emerging. The therapeutic index of brachytherapy needs to be considered, and the best sequencing of combined modalities needs to balance efficacy and toxicities. Additionally novel targeted therapies show promise, and further studies are needed to determine the appropriate use of these new agents. Management of endometrial cancer will continue to evolve as clinical trials continue to answer unsolved clinical questions.

## 1. Epidemiology of Endometrial Cancer

Endometrial cancer is the most common gynecologic malignancy in the United States and the fourth most common cancer in women, comprising 6% of female cancers. Only breast, lung, and colon cancers have higher incidence rates. The American Cancer Society estimated that there were 47,130 new cases of endometrial cancer and 8,010 deaths from endometrial cancer in 2012 [1]. Based on 2004–2008 Surveillance Epidemiology and End Results (SEER) data on endometrial cancer, the age-adjusted incidence rate is 23.9 per 100,000 women per year, and the age-adjusted death rate is 4.2 per 100,000 per year [2]. In the United States, the lifetime risk of developing endometrial cancer is 3%. Excluding women who have had a hysterectomy, 6% of women are diagnosed with endometrial cancer in their lifetime [3, 4]. Rising life expectancy and increasing rates and severity of obesity have contributed to the increasing incidence of endometrial cancer [5]. The National Health and Nutrition Examination Survey (NHANES) in 2009-2010 reported that 36% of adult females in the United States are obese [6].

While the absolute number of estimated new cases of endometrial cancer each year is similar between developed and developing countries, it occurs in a higher percentage of the population in developed countries. The developing world accounts for nearly 80% of the world's population but only about half of endometrial cancer cases [7]. Specifically, the International Agency for Research on Cancer through the GLOBOCAN series estimated 287,000 new cases of endometrial cancer and 74,000 deaths from endometrial cancer worldwide in 2008 [8]. There is a similar absolute distribution between developed and developing countries: GLOBOCAN estimated 142,000 new cases in developed countries and 145,000 new cases in developing countries, with 32,000 deaths in developed countries, in contrast to 41,000 deaths in developing countries [9]. The incidence rates of endometrial cancer are higher in Northern European and industrialized countries than in developing countries [3].

The incidence and 5-year survival rates of endometrial cancer also vary by race. The incidence of endometrial cancer in Caucasian women has remained stable, while the incidence in African American women has increased 2% per year. The death rate from endometrial cancer has remained both stable and disparate in both Caucasian and African American women. The relative 5-year survival in Caucasians is 84% in contrast to 60% in African Americans including all stages [2]. Overall, the 1-year survival rate is 92%, and the 5-year survival rate is 82%. Most endometrial cancers are diagnosed at early stage and have over 95% five-year survival rates (Table 1) [2].

Endometrial cancer is a diagnosis of older women, with a median age at diagnosis of 61 years. Over half of endometrial cancers are diagnosed in women who are 50 to 69 years old, and 32% of endometrial cancers are diagnosed between ages 55 and 64 [2].

Most endometrial cancers are adenocarcinomas and separated into type I and type II endometrial cancers based on clinical, pathologic, and molecular characteristics (Table 2) [3]. Grade 3 endometrioid adenocarcinomas have a propensity to behave as aggressively as type II tumors, which leads to controversy about how to classify them [9].

## 2. Risk Factors for Endometrial Cancer

### 2.1. Lifestyle and Behavioral Factors

Women exposed to unopposed estrogen are at risk for developing endometrial cancer. Increasing BMI significantly increases the risk for developing endometrial cancer (RR  1.59–2.89) with a higher relative risk for endometrial cancer-related death of 2.53 for obese women (BMI  30–34.9 kg/m^2^) and of 6.25 for morbidly obese women (BMI > 40 kg/m^2^) [10]. Multiple mechanisms explain the elevated endometrial cancer risk in obese women. Obesity increases the conversion of androstenedione to estrone by aromatase in adipose tissue. Obesity also leads to insulin resistance and decreased serum hormone binding globulin with a resulting increase in unbound biologically active estrogen and an increased inflammatory response [10, 11]. Occupations that are sedentary independently increase the risk of endometrial cancer by 28% [12]. A high-fat diet and diabetes (RR 3) are additional risk factors for endometrial cancer.

### 2.2. Reproductive and Menstrual History

Risk factors for endometrial cancer related to the reproductive and menstrual cycle include early menarche (before 12 years) (RR 1.5–2), late menopause (after 55 years) (RR 2-3), more lifetime menstrual cycles, nulliparity (RR 3), and infertility [13]. Similarly, pregnancy decreases the time that a woman menstruates, and duration of full term pregnancies creates a 22% per year cancer risk reduction [13]. The impact of duration of menstruation on endometrial cancer risk is likely multifactorial. Incessant menstruation from early menarche to late menopause in combination with nulliparity may lead to repetitive turnover of the cells of the endometrial lining, increasing the probability for sporadic DNA replication errors and consequent mutations in *PTEN* and *p53* [3]. Over 40% of type I endometrial cancers have a loss of *PTEN* and an activation of the PI3K/AKT/mTOR pathway [10, 14].

### 2.3. Genetic Conditions

Hereditary nonpolyposis colorectal cancer (HNPCC) is an autosomal dominant disorder, diagnosed by the Amsterdam criteria and resulting primarily from mutations in *MLH1* or *MSH2*. The lifetime risk of endometrial cancer is 40–60% in women with HNPCC. Cowden Syndrome, an autosomal dominant disorder characterized by multiple noncancerous hamartomas, is primarily caused by mutations in the *PTEN* gene. Five to 10% of women with Cowden Syndrome develop endometrial cancer [15].

### 2.4. Cancer and Precancer

Fifteen to 20% of granulosa-theca cell ovarian tumors and 30% of endometrioid ovarian cancers are associated with endometrial cancer. Other risk factors include a 10-fold increased risk with a family history of endometrial cancer at age younger than 50 years, personal history of breast or ovarian cancer, prior pelvic radiation, and endometrial hyperplasia [5, 10]. One percent of women with simple hyperplasia without atypia, 3% of women with complex hyperplasia without atypia, 8% of women with simple atypical hyperplasia, and 30–40% of women with complex atypical hyperplasia develop endometrial cancer.

### 2.5. Polycystic Ovarian Syndrome

Women with polycystic ovarian syndrome (PCOS) experience chronic anovulation with unopposed estrogen, leading to a 4-fold increased risk of developing endometrial cancer when compared to the general population, with an over two-fold increased risk when adjusted for BMI [10].

### 2.6. Use of Estrogen-Only Hormone Therapy

Women who take estrogen-only hormone therapy are at increased risk for developing endometrial cancer; progestins counter the effects of estrogen on the endometrial lining. The increased risk of endometrial cancer using estrogen-only hormone therapy is most pronounced in nonobese women [16].

### 2.7. Impact of Medications and Environment

The rate of endometrial cancer in women who take tamoxifen is 2-3 per 1000 women per year, and raloxifene is 1.25 per 1000 women per year. Talcum powder use has been shown to be associated with endometrial cancer. This may be due to increased inflammation with lower levels of antiMUC1 antibodies, activation of cytokines and macrophages, increased release of reactive oxygen species, increased cell turnover, and increased risk for DNA damage [10, 17].

## 3. Protective Factors against the Development of Endometrial Cancer

Oral contraceptives, physical activity, multiparity, and nonhormonal intrauterine device (IUD) use protect against endometrial cancer [10, 13].

### 3.1. Oral Contraceptives

Oral contraceptives decrease the risk of endometrial cancer by up to 50%. The duration of oral contraceptive use impacts the risk reduction, and that risk reduction is maintained for 10 years following discontinuation of oral contraceptives [10].

### 3.2. Physical Activity

Physical activity reduces endometrial cancer risk by 33–39%, an effect that is more pronounced in obese women [10, 12, 18]. Although physical activity reduces the risk for endometrial cancer, the Centers for Disease Control report that 49% of the US population does not engage in the recommended level of physical activity [19]. Increased insulin sensitivity, decreased body fat, and decreased circulating estrogen levels are possible explanations for the mechanism of the risk-reducing effects of physical activity on endometrial cancer risk.

### 3.3. Possible Protective Factors, Associations, and Areas for Future Study

Studies are inconclusive on the impact of hormonal IUDs, bariatric surgery, metformin, breastfeeding, and tubal sterilization on endometrial cancer risk. The levonorgestrel IUD has been shown to reverse complex atypical hyperplasia in multiple studies and may exert a protective effect against developing endometrial cancer, but more studies are needed to determine the impact of hormonal IUDs on endometrial cancer risk [10]. Metformin inhibits aromatase and therefore has the potential to exert a protective effect against endometrial cancer [10]. The impact of breastfeeding and duration of lactation on endometrial cancer risk is debated. Studies show a decreased risk of endometrial cancer with breastfeeding that is directly proportional to the duration of lactation; this risk reduction decreases with time, and lactation has no effect on endometrial cancer risk after age 50 [13, 20]. There is also debate about whether or not bariatric surgery and tubal sterilization decrease endometrial cancer risk [3, 21, 22]. Similarly, tobacco use is associated with a decreased risk of endometrial cancer. This may be because of the antiestrogenic effect of smoking, decreased BMI, or earlier menopause; the effect is most pronounced in current smokers and postmenopausal women [23].

### 3.4. Pathologic Associations with a Decreased Endometrial Cancer Risk

A history of bone fractures is associated with a lower risk of endometrial cancer likely because of the prolonged hypoestrogenic state that frequently leads to fractures. Systemic lupus erythematosus is also associated with a decreased risk for endometrial cancer (OR 0.71), possibly because these women tend to start menopause at a younger age [3, 24].

## 4. Endometrial Cancer Staging Revisions

In 2009, the International Federation of Gynecologists and Obstetricians (FIGO) revised the staging for endometrial cancer for the first time since the initial surgical staging in 1988. The 2009 FIGO staging for endometrial cancer now has separate staging systems for the 97% of epithelial carcinomas and the 3% of uterine sarcomas (Table 3). The notable changes are the combination of stages IA and IB into IA, encompassing superficial disease and disease with <50% myoinvasion, the elimination of stage IIA or cervical glandular involvement, the removal of peritoneal cytology, and the subdivision of stages IIIC into IIIC1 with positive pelvic nodes and IIIC2 with positive para-aortic nodes. Overall, the revisions in the 2009 staging appear to correlate more precisely with prognosis than the 1988 staging system. Formerly, survival had been better for IIA than IC, and currently all stage I cancers have improved survival over stage II cancers. Stages IIIC1 and IIIC2 also differ in prognosis. Whether or not peritoneal cytology is an independent prognostic factor is debated, but currently cytology has been removed from the staging system yet is still reported. There is also debate about the optimal extent of staging, specifically with regard to which patients need a lymphadenectomy and how aggressive the lymphadenectomy should be [25–27]. Sentinel lymph node biopsy in early-stage endometrial cancer may avoid the morbidity of a more extensive lymph node dissection while providing prognostic significance that could influence treatment decisions [27, 28].

## 5. Endometrial Cancer Presentations and Screening

Most women with endometrial cancer present with abnormal uterine bleeding or postmenopausal bleeding and are therefore diagnosed at an early stage [5]. Other common presenting symptoms include pain with urination, dyspareunia, pelvic pain, vaginal discharge, and weight loss [1]. Over 95% of women diagnosed with endometrial cancer present with symptoms. The less than 5% of women diagnosed without symptoms are diagnosed through workup of abnormal Pap smear, abnormal finding on imaging, or as an incidental finding on pathology at time of hysterectomy. Although postmenopausal bleeding is the most common presenting symptom, only 10% of women with postmenopausal bleeding have endometrial cancer. Pipelle endometrial biopsy (EMB) is the preferred method for evaluation of abnormal uterine bleeding because of its high sensitivity, low cost, and low morbidity in comparison to other sampling devices. The false negative rate of Pipelle EMB increases when less than 50% of the endometrial cavity is affected by disease [29]. Postmenopausal women with an endometrial stripe by transvaginal ultrasound (TVUS) less than 4 or 5 mm are at low risk for endometrial cancer [30]. Saline infusion sonohysterography has a higher sensitivity and specificity for detection of endometrial polyps but with potential increased patient discomfort, lack of tissue diagnosis, and higher costs, making it an alternative but not preferred method for evaluation of abnormal uterine bleeding [31]. Women who continue to be symptomatic should have a fractional dilation and curettage (D and C) with or without hysteroscopy [5]. Both saline infusion sonohysterography and hysteroscopy have theoretical risks of dissemination of tumor cells.

Routine screening for endometrial cancer is not recommended in the general population. In women with HNPCC Syndrome, the American Cancer Society recommends annual screening with endometrial biopsy and/or transvaginal ultrasound starting at age 35 [1], and the National Comprehensive Cancer Network (NCCN) recommends that all women with HNPCC undergo yearly EMBs until hysterectomy and bilateral salpingo-oophorectomy after completion of childbearing [5]. Asymptomatic women who are taking tamoxifen should not be routinely screened. Women on tamoxifen should be evaluated if they develop vaginal bleeding with an EMB or D and C [32]. Patients who are undergoing endometrial ablation should have an EMB prior to ablation. No routine screening is recommended in women with Cowden Syndrome.

## 6. Endometrial Cancer Treatment

Treatment of endometrial cancer is on one level very straightforward and yet on another level evolving and fraught with controversies. The mainstay of treatment for endometrial cancer is surgery including total hysterectomy, peritoneal cytology, and bilateral salpingo-oophorectomy followed by intraoperative staging as indicated. Adjuvant therapy is based upon final stage, patient characteristics, and peritoneal cytology status.

### 6.1. Role of Lymphadenectomy in Endometrial Cancer

The role of pelvic and para-aortic lymphadenectomy in endometrial cancer is controversial. Experts debate whether lymphadenectomy is simply diagnostic or also therapeutic and whether or not there is benefit to node dissection for all or only a selected group of patients. The Gynecologic Oncology Group surgicopathology study (GOG 33) identified multiple prognostic factors that impact the likelihood of nodal disease and overall survival (OS). Endometrial cancer is now categorized into low, intermediate and high-risk disease based on tumor size, grade, extent of myometrial invasion, cervical stromal involvement, lymphovascular space invasion (LVSI), and increased age [33–36].

Multiple randomized controlled trials have shown no OS benefit from lymphadenectomy in early-stage, low-risk disease. Panici et al. in a randomized trial of over 500 patients with stage I endometrial cancer reported no difference in disease-free survival (80% vs. 82%) or OS (90% vs. 86%) between the lymphadenectomy and no lymphadenectomy groups [37]. Although the lymphadenectomy group in comparison to the no lymphadenectomy group had a higher rate of upstaging, they also had a higher complication rate (*P* = 0.001). Similarly A Study in the Treatment of Endometrial Cancer (ASTEC) trial from the United Kingdom examined 1400 women, with endometrial cancer confined to the uterus on preoperative assessment, and demonstrated no OS benefit from pelvic lymphadenectomy in early-stage endometrial cancer with a hazard ratio for OS of 1.04 and recurrence-free survival of 1.25 in favor of no lymphadenectomy in comparison to lymphadenectomy [38]. Both of these trials were performed in low-risk populations; they were underpowered and have been criticized for their study design. The second randomization to adjuvant radiation in the ASTEC trial has led many to conclude that by attempting to assess the impacts of both lymphadenectomy and radiation on survival, the authors were not able to assess either condition [39]. Additionally critics of the ASTEC trial note the baseline differences in the lymphadenectomy and no lymphadenectomy arms as well as the inadequacy of lymph node dissection as weaknesses of the trial [39]. In contrast to the ASTEC and Italian trial findings, Chan et al. in a Surveillance, Epidemiology, and End Results (SEER) study of 12,333 patients recognized improved 5-year disease-specific survival with lymphadenectomy in stage IB grade 3 and higher patients when patients were matched by stage and those with and without lymphadenectomy were compared (*P* < 0.001) [40].

One approach to this controversy of the value of lymphadenectomy has been the development of algorithms for patient selection such as the Mayo Clinic criteria (Table 4) [41]. Although an analysis showed increased cost and morbidity without survival benefit with lymphadenectomy in low-risk patients as defined by the Mayo criteria, these criteria depend on intraoperative frozen pathology, and frozen and final pathology discrepancies vary by institution, limiting the generalizability of these data [42].

Although these data demonstrate a lack of therapeutic benefit in early-stage endometrial cancer, Bristow et al. in retrospective study of 40 patients with stage IIIC endometrial cancer showed a statistically significant disease-specific survival benefit of 37.5 months versus 8.8 months (*P* = 0.006) from debulking macroscopic adenopathy with node-positive, advanced disease [43]. In addition to this therapeutic benefit, lymph node dissection identifies patients who do not need adjuvant therapy or who can receive less aggressive adjuvant therapy. Proponents of routine lymph node dissection debate the extent of lymphadenectomy as well as the criteria for determining if a lymphadenectomy is adequate. Fotopoulou et al. concluded that lymphadenectomy should be extended superior to the inferior mesenteric artery (IMA) to the level of the renal veins after finding that, in intermediate and high-risk node-positive patients, 76% of patients will have positive para-aortic nodes, with LVSI and incomplete tumor resection being the largest predictors of positive nodal status [44]. Mariani et al. reported in a prospective assessment of lymph node metastases that 16% of patients had isolated positive para-aortic lymph node, and, of patients with para-aortic lymph node involvement, 77% had positive nodes above the IMA [41].

Those in opposition to routine lymphadenectomy have concerns about the short- and long-term complications especially from lymphocyst and lymphedema. Rates of lymphedema and lymphocyst range from 1.2 to 3.1% [36, 45–47]. Given the potential morbidity of lymphadenectomy, a prospective study of 115 patients examined the efficacy of sentinel lymph node mapping using isosulfan or methylene blue dye and technetium-99 in endometrial cancer. An overall 85% detection rate was found with sentinel lymph node mapping when followed by confirmatory regional lymph node dissection. Rate of successful mapping improved from 77% to 94% after an individual completed 30 cases [48]. Sentinel lymph node mapping requires further validation prior to routine use.

### 6.2. Mode of Primary Surgical Treatment and Alternative Primary Management of Endometrial Cancer

Although traditionally surgical management of endometrial cancer was performed via laparotomy, current management of endometrial cancer incorporates minimally invasive approaches when feasible, which offer the least morbid, optimal treatment option for these women who often have significant comorbidities. The GOG LAP-2 randomized over 2600 patients to laparoscopy versus laparotomy. The laparoscopic group had fewer postoperative complications (14% vs. 21%, *P* < 0.0001) and shorter hospital stays over 2 days (52% vs. 94%, *P* < 0.0001) but longer operating times (204 minutes vs. 130 minutes, *P* < 0.001). A secondary survival analysis demonstrated similar recurrence risk (11% vs. 10% at 3 years), which did not meet the protocol-specified definition of noninferiority, and OS (90% at 5 years in both groups) [49, 50].

Similar to LAP-2, other studies have demonstrated benefits to minimally invasive techniques, including robotic-assisted surgical management of endometrial cancer, making it an acceptable alternative to laparoscopy [51]. Importantly, a study of 2,464 women undergoing minimally invasive hysterectomy for endometrial cancer found no difference in morbidity but increased cost with robotic hysterectomy compared to laparoscopic hysterectomy [52].

Total vaginal hysterectomy is also a reasonable approach to management of early endometrial cancer but is limited in exploration of the abdominal cavity, lymph node dissection, peritoneal washings, and further staging as indicated.

With the overall favorable prognosis for early-stage, low-grade, and type I histology endometrial cancer, fertility preservation is a temporizing treatment option for women who understand and accept the risks. In a SEER database study of over 3200 premenopausal women with stage I endometrial cancer, ovarian preservation was not associated with increased cancer-related or overall survival difference [53]. In a prospective, multi-institution study in Japan, women desiring fertility less than 40 years of age with presumed stage IA endometrial cancer or atypical endometrial hyperplasia were treated with primary medroxyprogesterone acetate and low-dose aspirin. Complete response rates were 55% and 82% for endometrial cancer and atypical hyperplasia, respectively, with 47% recurrence rate at 3 years [54].

In presumed stage I-II patients who are medically inoperable, primary radiation therapy offers a feasible alternative to primary surgery with a 16% recurrence rate and a 3.4 times higher likelihood of death from a cause other than cancer [55].

### 6.3. Adjuvant Treatment for Endometrial Cancer

Optimal adjuvant treatment for endometrial cancer is controversial. Four randomized controlled trials of early-stage endometrial cancer patients, the Norwegian trial, Post Operative Radiation Therapy in Endometrial Carcinoma (PORTEC-1), GOG-99, and ASTEC/EN 5, showed improved locoregional control but no OS benefit with radiation therapy (Table 5) [56–59]. Additionally gastrointestinal (GI) toxicity was higher in the external beam radiation therapy group, and adjuvant radiotherapy had a negative impact on quality of life [60].

In contrast to the four randomized controlled trials showing no survival benefit with adjuvant radiation therapy in early endometrial cancer, in an analysis of over 21,000 women from the Surveillance, Epidemiology, and End Results (SEER) database, stage IC grades 1 and 3 showed improved overall and relative survival from adjuvant radiation with hazards ratios of 0.44 (*P* < 0.001) and 0.72 (*P* = 0.009), respectively [61]. Despite randomized controlled trials showing no survival benefit with adjuvant radiation in early endometrial cancer, these data elucidate the importance of identifying an early-stage, high-risk population that might benefit from adjuvant therapy. GOG-99 identified this high-intermediate risk group of patients based on clinical and pathologic features (Table 6) [58]. Adjuvant radiation therapy in this high-intermediate risk group decreased recurrence risk. Additionally, the high-intermediate risk subgroup treated with adjuvant radiation therapy appeared to have a survival advantage (RH 0.73, 90% CI 0.43–1.26). Unfortunately, because of the predominance of low-risk patients and the fact that so many patients died of causes other than endometrial cancer in both arms, the study was underpowered both for overall survival and subgroup analysis of high versus low intermediate risk. As a result, adjuvant radiation therapy may be considered in high-intermediate risk but not low-risk patients. NCCN presents guidelines for management of early-stage and advanced local endometrial cancer (Tables 7 and 8) [62]. Note that current investigation, as well as these guidelines, has advocated consideration of chemotherapy for high-risk, local disease.

The long-term morbidity of whole pelvic radiotherapy and the frequency vaginal cuff recurrences prompted study of vaginal brachytherapy in comparison to pelvic radiotherapy. PORTEC-2 demonstrated similar locoregional recurrence and OS but decreased GI toxicity (13% vs. 54%) with adjuvant vaginal brachytherapy in comparison to external beam radiation therapy [63]. Based on these findings and the low morbidity of vaginal brachytherapy, vaginal brachytherapy is frequently used for adjuvant treatment in intermediate-risk patients.

Chemotherapy has emerged as an important component of adjuvant treatment due to the recognition that many patients with high-risk disease will have a component of the recurrence outside the pelvis. Multiple trials compare chemotherapy with radiation with combination treatment (Table 9) [64–68]. GOG 122 demonstrated the value of chemotherapy in stage III-IV patients with improved survival with doxorubicin and cisplatin in comparison to whole abdominal radiotherapy, PFS  HR = 0.71  (*P* < 0.01); OS  HR = 0.68  (*P* < 0.01) [69]. Adding paclitaxel to this chemotherapeutic regimen did not improve survival and demonstrated greater toxicity [68]. Carboplatin and paclitaxel have demonstrated efficacy in adjuvant treatment for advanced stage disease with minimal toxicity in retrospective analysis [70]. In stages III and IV patients with extrauterine disease, chemotherapy is the treatment of choice. Although no prospective trials have examined the optimal sequencing of chemotherapy and radiation, a multicenter retrospective cohort of advanced stage endometrial cancer showed overall and progression free-survival benefit with the sandwich technique of chemotherapy followed by radiation followed by chemotherapy [71].

Although the role of adjuvant chemotherapy for advanced stage endometrial cancer is standard, the role of adjuvant chemotherapy in early endometrial cancer is controversial. Randomized trials in Europe examined adjuvant treatment in surgically treated women with stages I, II, and III endometrial cancer who had no residual tumor. The addition of sequential chemotherapy to adjuvant radiotherapy led to a reduced risk of both relapse and death and improved cancer-specific survival (*P* = 0.01) versus adjuvant radiotherapy alone (HR = 0.74) [67]. Given these encouraging data on the role of adjuvant chemotherapy in high-risk endometrial cancer, two trials, examining the role of chemotherapy in high-risk, uterine-confined endometrial cancer, are currently ongoing. The PORTEC-3 trial is comparing chemoradiation with radiotherapy alone in high-risk stages I, II, and III endometrial cancer, and GOG-249 is comparing whole pelvic radiation with vaginal brachytherapy and carboplatin and paclitaxel in high-intermediate risk endometrial cancer.

For isolated vaginal cuff recurrence, radiation is the treatment of choice [72]. Chemotherapy or progestational agents are treatment options for recurrent endometrial cancer that is not localized. Surgery is an option for patients who have already had radiation treatment. Hormonal therapy with progestins or tamoxifen has also demonstrated efficacy in recurrence. Multiple GOG trials have examined hormone therapy in endometrial cancer (Table 10) [73]. Additionally many novel biologic therapies are under investigation; temsirolimus, an mTOR inhibitor, has activity in advanced stage and recurrent endometrial cancer [74].

## 7. Conclusions

In conclusion, although endometrial cancer management is in many ways straightforward, many controversies still require further debate and investigation (Table 11). Given that patients frequently present with symptoms and providers are able to assess disease with EMB and D and C, most patients are diagnosed with early-stage disease and have a favorable prognosis. As a result, many general practitioners perform hysterectomies for complex atypical hyperplasia despite its 25–40% rate of concurrent endometrial cancer and generalists' lack of training in lymph node dissection. One of the primary benefits of lymphadenectomy is identification of patients who can either avoid or receive less aggressive adjuvant therapy. The role and extent of lymphadenectomy especially in low-risk patients are debated. The Mayo criteria are one approach to providing guidance for determination of low-risk patients in whom to omit lymphadenectomy, although these criteria rely on intraoperative frozen pathology, which may be discrepant from final pathology. The change in surgical staging correlates more closely with prognosis and removes peritoneal cytology from the staging system. The importance of cytology is unknown, and currently data are being prospectively collected for evaluation. Adjuvant treatment of endometrial cancer is changing with debates about the roles of radiation in intermediate-risk patients and chemotherapy in high-risk patients. Additionally targeted therapies such as mTOR inhibitors show promise in endometrial cancer. Management of endometrial cancer will continue to evolve as studies begin to answer these controversial questions.

## Figures and Tables

**Table 1:** Endometrial cancer stage distribution and five-year survival.

Stage	Stage distribution*	Five-year survival
Local disease	68%	96%
Regional disease	20%	67%
Distant disease	8%	16%

*Based on SEER 2001–2007 data. Total not 100% since stage of disease is sometimes unknown.

**Table 2:** Type I and II endometrial cancers.

	Type I endometrial cancers	Type II endometrial cancers
Hormonal impact	Estrogen dependent	Estrogen independent
Histology	Endometrioid adenocarcinomas	Clear-cell, serous, uterine carcinosarcomas
Patient population	Younger, obese, perimenopausal	Older, thin, postmenopausal
Distribution	85%	15%
Prognosis	Better differentiated	More aggressive, proportionally higher mortality
Genetic mutations	Kras, PTEN, MLH1	p53, erbB2

**Table 3:** 2009 FIGO endometrial cancer staging.

Stage	
I	Tumor confined to the corpus uteri.
IA	No or less than half myometrial invasion.
IB	Invasion equal to or more than half of the myometrium.
II	Tumor invades cervical stroma but does not extend beyond the uterus.
III	Local and/or regional spread of the tumor.
IIIA	Tumor invades the serosa of the corpus uteri and/or adnexae.
IIIB	Vaginal and/or parametrial involvement.
IIIC	Metastases to pelvic and/or para-aortic lymph nodes.
IIIC1	Positive pelvic nodes.
IIIC2	Positive para-aortic lymph nodes with or without positive pelvic lymph nodes.
IV	Tumor invades bladder and/or bowel mucosa, and/or distant metastases.
IVA	Tumor invasion of bladder and/or bowel mucosa.
IVB	Distant metastases, including intra-abdominal metastases and/or inguinal lymph nodes.

**Table 4:** Mayo criteria for omission of lymphadenectomy in surgical management of endometrial cancer.

	Omit lymphadenectomy if no disease beyond the uterine corpus AND
	(1) Endometrioid grade 1 or 2, myometrial invasion ≤50%, and tumor diameter ≤2 cm OR
	(2) Endometrioid and no myometrial invasion independent of grade and tumor diameter

**Table 5:** Randomized trials of adjuvant radiation therapy in early-stage endometrial cancer.

Trial	Norwegian trial	PORTEC-1	GOG-99	ASTEC/EN 5
Authors	Aalders et al.	Creutzberg et al.	Keys et al.	ASTEC/EN 5 Study Group et al.
Endometrial cancer stage	I	I, grade 1, 2, 3	IB, IC, II	I, IIA
Number of patients	540	715	392	905
Adjuvant treatment	Vaginal brachytherapy (VBT) versus VBT and pelvic radiation therapy (RT)	Observation versus RT	Observation versus RT	Observation versus RT
Vaginal and pelvic recurrence	7% versus 2% (*P* < 0.01)	14% versus 4% (*P* < 0.001)	12% versus 3%	6% versus 3%
Overall survival	89% versus 91%	85% versus 81% (*P* = 0.31)	86% versus 92% (*P* = 0.557)	84% versus 84%
Extent of staging	Staging not mandated	Staging not mandated	Staging mandated	50% staged

**Table 6:** High-intermediate risk endometrial cancer patients.

	Age ≥70 with 1 risk factor
	Age ≥50 with 2 risk factors
	Age ≥18 with 3 risk factors
	Risk factors: grade 2/3 tumors, LVSI, outer 1/3 myometrium

**Table 7:** NCCN guidelines for adjuvant treatment of early-stage endometrial cancer.

Stage	Adverse risk factors^a^		Grade	
		1	2	3
IA	Not present	Observe	Observe or brachytherapy	Observe or brachytherapy
	Present	Observe or brachytherapy	Observe or brachytherapy ± WPRT (category 2B)	Observe or brachytherapy ± WPRT
IB	Not present	Observe or brachytherapy	Observe or brachytherapy	Observe or brachytherapy ± WPRT
	Present	Observe or brachytherapy ± WPRT	Observe or brachytherapy ± WPRT	WPRT ± brachytherapy ± chemotherapy (category 2B) or observe (category 2B)

^a^Risk factors: age >60; lymphovascular space invasion (LVSI); Tumor size >2 cm; lower uterine (cervical/glandular) involvement.

WPRT: whole pelvic radiation therapy.

**Table 8:** Adjuvant treatment of advanced local endometrial cancer.

Stage		Grade	
	1	2	3
II	Brachytherapy ± WPRT	WPRT + brachytherapy	WPRT ± brachytherapy ± chemotherapy (category 2B)
IIIA	Chemotherapy ± WPRT or tumor-directed RT ± chemotherapy or WPRT ± brachytherapy	Chemotherapy ± WPRT or tumor-directed RT ± chemotherapy or WPRT ± brachytherapy	Chemotherapy ± WPRT or tumor-directed RT ± chemotherapy or WPRT ± brachytherapy

WPRT: whole pelvic radiation therapy; RT: radiation therapy.

**Table 9:** Adjuvant treatment in endometrial cancer trials: radiation versus chemotherapy versus combination.

Study	*N*	Stage	Drug regimen	5-year PFS (%)	5-year OS (%)
Randall GOG 122	386	III/IV	AP versus WAI	50 38	55 42
Maggi	340	I-III	CAP versus PRT	63 63	66 60
Susumu JGOG	385	I-III	CAP versus PRT	82 84	85 87
Hogberg	372	I-III	Various versus PRT	79 72	88 78
Homesely GOG 184	552	III/IV	PRT + AP PRT + TAP	62 (3 year) 63 (3 year)	NS

AP: doxorubicin-cisplatin;

WAI: Whole-abdominal irradiation;

CAP: cyclophosphamide-doxorubicin-cisplatin;

PRT: Pelvic radiation therapy;

TAP: paclitaxel-doxorubicin-cisplatin.

**Table 10:** GOG trials of hormone therapy in endometrial cancer.

GOG study and dosing	RR (%)	PFS (months)	OS (months)	DOR (months)
153 MA 80 mg BID × 3 weeks alternating with T 20 mg BID × 3 weeks	27%	2.7 months	14.0 months	28 months
121 high dose MA 800 mg daily	24%	2.5 months	7.6 months	8.9 months
81 MA high dose 1000 mg daily versus low dose 200 mg daily	15% high dose 25% low dose	2.5 months 3.2 months	7.0 months 11.1 months	NR
119 T 200 mg BID + MPA 100 mg BID intermittently weekly	33%	3.0 months	12.8 months	NR
81F T 20 mg BID	10%	1.9 months	8.8 months	NR

MA: megastrol acetate; T: tamoxifen; MPA: medroxyprogesterone acetate; RR: response rate; PFS: progression free-survival (median); OS: overall survival (median); DOR: duration of response (median); NR: not reported.

**Table 11:** Selected treatment controversies in endometrial cancer.

Question/controversy	Comment
Role of lymphadenectomy in low risk	ASTEC trial did not support but results not universally accepted
Prognostic value of peritoneal cytology	Removed as a staging variable recently but still to be collected and reported
Role of radiation in intermediate risk	Local control improved but no overall survival benefit
Best adjuvant for high-risk disease	Recent data supports carboplatin/paclitaxel combination
Should targeted therapies be utilized	Further clinical and basic science research required; mTOR inhibtors promising
